# The Effect of TiO_2_ Nanoparticles on the Composition and Ultrastructure of Wheat

**DOI:** 10.3390/nano11123413

**Published:** 2021-12-16

**Authors:** Maria-Loredana Soran, Ildiko Lung, Ocsana Opriș, Otilia Culicov, Alexandra Ciorîță, Adina Stegarescu, Inga Zinicovscaia, Nikita Yushin, Konstantin Vergel, Irina Kacso, Gheorghe Borodi

**Affiliations:** 1 National Institute for Research and Development of Isotopic and Molecular Technologies, 67-103 Donat, 400293 Cluj-Napoca, Romania; loredana.soran@itim-cj.ro (M.-L.S.); ildiko.lung@itim-cj.ro (I.L.); ocsana.opris@itim-cj.ro (O.O.); alexandra.ciorita@itim-cj.ro (A.C.); adina.stegarescu@itim-cj.ro (A.S.); irina.kacso@itim-cj.ro (I.K.); gheorghe.borodi@itim-cj.ro (G.B.); 2 Joint Institute for Nuclear Research, 6 Joliot-Curie, 1419890 Dubna, Russia; inga@jinr.ru (I.Z.); ynik_62@mail.ru (N.Y.); verkn@mail.ru (K.V.); 3 National Institute for Research and Development in Electrical Engineering ICPE-CA, 313 Splaiul Unirii, 030138 Bucharest, Romania; 4 Department of Molecular Biology and Biotechnologies, Faculty of Biology and Geology, Babeș-Bolyai University, 5-7 Clinicilor, 400006 Cluj-Napoca, Romania; 5 Horia Hulubei National Institute for Physics and Nuclear Engineering, 407 Atomistilor, 077125 Magurele, Romania

**Keywords:** wheat, TiO_2_, nanoparticles, bioactive compounds, antioxidant capacity, elemental content

## Abstract

The present work aims to follow the influence of TiO_2_ nanoparticles (TiO_2_ NPs) on bioactive compounds, the elemental content of wheat, and on wheat leaves’ ultrastructure. Synthesized nanoparticles were characterized by X-ray diffraction (XRD), Fourier transform infrared (FT-IR) spectroscopy, and transmission electron microscopy (TEM). The concentration of phenolic compounds, assimilation pigments, antioxidant capacity, elemental content, as well as the ultrastructural changes that may occur in the wheat plants grown in the presence or absence of TiO_2_ NPs were evaluated. In plants grown in the presence of TiO_2_ NPs, the amount of assimilating pigments and total polyphenols decreased compared to the control sample, while the antioxidant activity of plants grown in amended soil was higher than those grown in control soil. Following ultrastructural analysis, no significant changes were observed in the leaves of TiO_2_-treated plants. Application of TiO_2_ NPs to soil caused a significant reaction of the plant to stress conditions. This was revealed by the increase of antioxidant capacity and the decrease of chlorophyll, total polyphenols, and carotenoids. Besides, the application of TiO_2_ NPs led to significant positive (K, Zn, Br, and Mo) and negative (Na, Mn, Fe, As, Sr, Sb, and Ba) variation of content.

## 1. Introduction

Nanoparticles (NPs), including silver, silica, aluminum, zinc oxide, copper, carbon nanotubes or TiO_2_, are increasingly manufactured and implemented in various products. NPs are produced on a large scale from a large variety of bulk materials with applications in many areas including medicine and agriculture [1,2]. The application of use of nanoparticles in agriculture has increased due to the possibility of using them as fertilizers or in the composition of plant protection products. As a consequence, it is important to know their effect on plant life [3]. Despite of the great potential in agricultural use of nanoparticles, there is a lack of studies about the toxicity of these nanoparticles in environments such as soil or in the growth of vegetables. Food security has become a high-priority concern for sustainable global development both quantitatively and qualitatively. The adverse effects of contaminants on crop quality can threaten human health and thus the subject is important [4,5,6,7].

Among all extensively used nanoparticles, TiO_2_ is of great interest to the scientific community due its versatility of use in a large variety of products (e.g., cosmetics, sunscreens, paints, and pigments). It is also used to improve the quality of the environment (e.g., water, soil, and air) [8,9,10,11].

Gottschalk et al. [12], in 2009, after a computer modeling assessment, demonstrated that a mass of 136 mg TiO_2_ NPs is present in one kg of sewage sludge, and if these sludges are used in agriculture areas, they can affect the soil and plant health for a long time. In addition, the presence of TiO_2_ NPs changed the bacterial community of activated sludge [13]. These can have a positive effect on plants if are present in a low concentration. Up to 60 mg kg^−1^ TiO_2_ NPs increased root and shoot length with a positive effect on total fresh and dry biomass. Wheat crops accumulated titanium in their roots which translocated to the leaves of the culture grown in soil enriched with TiO_2_ NPs. Their conclusion was that TiO_2_ NPs can have an inhibitory effect and cause cell damage at higher concentration (>60 mg kg^−1^) and more investigations will be necessary to study the effect of NPs on agriculture crops [12]. Rafique et al. [8], in 2014, following the experimental results, obtained the same conclusion. They used spherical-shaped TiO_2_ NPs 11.93–18.67 nm in size. The experimental results showed that the roots, shoot length, and biomass of wheat were significantly affected. Inhibitory and cell damage at treatments with higher concentrations of TiO_2_ NPs and positive effects at concentration levels up to 60 mg kg^−1^ were observed. These results suggest that further investigations should be made to determine the possible consequences and impacts of applying nanoparticles in wheat culture or other crops.

Another complementary study made by Florian Klingenfuss [14] in his thesis research, in 2014, presents a long-lasting experiment (84 days) on soil microcosm using TiO_2_ NPs with sizes 28 nm, 91 nm, and 100 nm. He evaluated the toxicity of these kinds of nanoparticles towards wheat and soil microorganisms with promising results at concentrations of 1, 100, and 1000 mg kg^−1^ soil. No side effects were detected after these experiments at the presented concentrations. The analysis of wheat grains indicated the presence of high concentrations of titanium, thus suggesting a translocation of NPs from the soil into grains. The microbial DNA analysis from nanoparticle-modified soil did not differ from the control [14].

Ma et al. and Nair et al. [15,16], in 2010, showed that crops grown in soil enriched in TiO_2_ NPs can accumulate nanoparticles in their roots can translocate to their seeds, leaves, and fruits. In addition, they demonstrated that a significant accumulation depends on the physical and chemical properties of the nanoparticles (chemical composition, shape, size, or agglomeration) and the plant species [15,16,17].

The scientific literature is still divided because some researchers show beneficial effects and others negative effects. For this reason, it is necessary to further study the long-term effect of nanomaterials, especially on wheat, because it is the most widely-grown crop worldwide [18,19,20].

Du et al. [21], in 2011, studied of the effect of TiO_2_ and ZnO NPs with size <100 nm on wheat growth and soil enzyme activities under field conditions. The nanoparticles reduced the biomass of wheat due to their toxicity effect. The authors presented results that demonstrated significant changes in the soil environment and important influences on soil enzyme activities resulting either from the presence of the TiO_2_ NPs or by the dissolution of the ions in soil in the case of ZnO NPs. They concluded that further experiments with NPs of various sizes, different species of plants, and different conditions of exposure are necessary, because nanoparticles were shown to be categorically toxic for the soil ecosystem.

Another important property of TiO_2_ NPs was reported by Satti et al. [22]. They used various concentrations of these nanoparticles (20, 40, 60, and 80 mg L^−1^) with size 40–65 nm, spherical shape, on the wheat culture affected by a fungi disease (*Bipolaris sorokiniana*). It was demonstrated that the exposure of plants at 40 mg L^−1^ reduced the disease without modification of wheat quality and yield. The morphological, physiological, and non-enzymatic metabolites parameters (e.g., leaf and root surface area, plant fresh, yield parameters, relative water content, chlorophyll content, or phenol and flavonoid content) were also monitored. The achieved promising results under biotic stress at 40 mg L^−1^ concentration of TiO_2_ NPs against fungal diseases and an improvement of the quality and yield of wheat were obtained.

Some research presents the beneficial potential of utilization of TiO_2_ NPs during the germination process. It was observed that using wheat seeds to soak in different concentrations of TiO_2_ NPs (0.0%, 0.025%, 0.05%, 0.1%, 0.2%, and 0.5%) stimulate wheat growth in normal germination stage. The best results were obtained at lower concentration (0.1%) with beneficial results in increasing vigor, root dry matter stress tolerance, dry matter stress tolerance index, plant height stress tolerance, root-length stress tolerance index, and pigment composition. At a higher concentration, 0.5% of TiO_2_ NPs, all parameters were suppressed [3]. Jaberzadeh et al. [23], in 2013, followed the influence of these kinds of nanoparticles on the seed gluten and starch contents of wheat under water deficit conditions, during one season of growth. They also used different concentrations (0.01%, 0.02%, and 0.03%) of TiO_2_ NPs and followed the same parameters as Shafea et al. (2017) [3]. The best results obtained at 0.02% nanoparticles increased almost all agronomic parameters, including gluten and starch content.

We can conclude that many aspects associated with the application of nanotechnology to agricultural activities are still unknown and there is not enough information on nanotoxi-cology in crops. Further studies will be necessary to clarify the beneficial or non-beneficial effect of nanoparticles in agronomic areas.

The main objective of this study consists of the evaluation of TiO_2_ NPs’ effect on the bioactive compounds and elemental content of wheat (*Triticum aestivum* L.) and the assessment of their impact on the ultrastructure of leaves. The novelty consists of determining the total polyphenols and the antioxidant activity evaluated by the DPPH method, simultaneously with a large number of chemical elements in wheat treated with TiO_2_ NPs through soil. In addition, we performed ultrastructural analysis of wheat leaves grown in this way.

## 2. Materials and Methods

### 2.1. Chemicals and Materials

For nanoparticles synthesis and wheat extracts analysis, titanium tetrachloride (TiCl_4_), Folin–Ciocalteu reagent, gallic acid, anhydrous carbonate 2,2-diphenyl-picrylhydrazyl (DPPH), and 6-hydroxy-2,5,7,8-tetramethylchroman-2 carboxylic acid (Trolox) were employed from Sigma-Aldrich, Schnelldorf, Germany, while the ethanol used for extracts obtaining was purchased from Chimopar, Bucharest, Romania. All chemicals used in the experiments were of analytical grade.

### 2.2. Nanoparticle Preparation and Characterization

TiO_2_ NPs were obtained from TiCl_4_ to which double-distilled water was added. The mixture was stirred for 30 min at room temperature, then heated to 150 °C and kept until the nanoparticles were formed. The nanoparticles were separated by centrifugation, washed with double-distilled water and ethanol, and dried in an oven at 65 °C.

The synthesized nanoparticles were structurally characterized by X-ray powder diffraction analysis using D8 Advance diffractometer equipped with Ge (111) monochromator in order to have only CuKα1 radiation, and LynxEye superspeed position detector. The aspect of the obtained nanoparticles was analyzed using scanning/transmission electron microscopy, STEM HITACHI HD2700 (from LIME-INCDTIM, Cluj-Napoca, Romania), cold field emission, operated at 200 kV, and coupled with a double EDX system, used to confirm the elemental distribution in the nanoparticles, and the size distribution was analyzed using Image J. FT-IR measurements were performed with a JASCO 6100 FT-IR spectrometer in the 4000–400 cm^−1^ spectral domain with a resolution of 4 cm^−1^ using the KBr pellet technique.

### 2.3. Plant Growth Conditions

In order to study the effect and the uptake of TiO_2_ NPs, autumn wheat (*Triticum aestivum* L.) was used. In this regard, 50 grains of wheat were sown at a depth of 1 cm in plastic pots (0.81 L, 13.5 cm in diameter) for 6 weeks. The pots contained 400 g of garden substrate with active humus and fertilizer (Agro, 50 L) for control, and other pots contained 400 g of garden substrate mixed with 120 mg TiO_2_ NPs. The plants were grown under controlled light (for 12 h from 24 h), humidity (60%), and temperature (day/night cycle of 20/10 °C) conditions.

Plant were watered with 80 mL of double-distilled water every 2 days.

### 2.4. Plant Tissues and Soil Analysis after Harvesting

#### 2.4.1. Assimilating Pigments Determination

Assimilating pigments extract was obtained by adding 20 mL of acetone to the minced leaves of wheat. The mixture was then stirred on a shaker for 30 min at 300 rpm at room temperature. After stirring, the mixture was centrifuged for 10 min and the supernatant was decanted. The operation was repeated with another 20 mL, and then 10 mL of acetone, the ratio of plant and acetone being 1:100. All three solutions were combined in the same bottle, and the extractions were performed in triplicate.

The pigments’ quantification from the plant extract was performed by UV-VIS spectroscopy, by recording the absorption spectra of the extracts in the wavelength range between 400 and 750 nm. The concentrations of chlorophyll a, chlorophyll b, and total carotenoids were calculated according to Lichtenthaler and Buschmann [24].

#### 2.4.2. Total Polyphenols Quantification

Polyphenolic extracts were obtained from 60% ethanol and milled leaves of wheat in a ratio of 1:15. The mixture was sonicated for 30 min at room temperature. The supernatant was separated by centrifugation for 10 min at 7000 rpm and stored at 4 °C until analysis.

The Folin–Ciocalteu method was used for total polyphenol content evaluation [25]. For this purpose, to a 10 mL graduated flask containing 5 mL of ultrapure water was added 1 mL of the extract and 0.5 mL of Folin–Ciocalteu reagent, and the contents were mixed. After 3 min, 1.5 mL Na_2_CO_3_ (5 g L^−1^), and double-distilled water till 10 mL were added. The mixture was maintained at 50 °C (in a water bath) for 16 min and after cooling to room temperature, their absorbances were read in relation to the blank sample (double-distilled water) at a wavelength of 765 nm. All measurements were taken in triplicate and mean values were calculated.

The total polyphenol concentration of the samples was calculated using a standard curve of gallic acid for the range of 0.002–0.8 mg mL^−1^.

#### 2.4.3. Antioxidant Capacity Determination of Polyphenolic Extracts

The antioxidant capacity was determined with a slightly modified method from Brand-Williams et al. [26], consisting of the reduction of 2,2-diphenyl-picrylylhydrazyl radicals (DPPH). Thus, to 3.9 mL of DPPH radical solution (0.0025 g/100 mL methanol) 0.01 mL of alcoholic extract was added, and after 10 min of rest in the dark, the absorbance of the mixture was measured at 515 nm relative to the control sample consisting of 3.9 mL methanol, to which was added 0.01 mL of extract.

#### 2.4.4. TEM Analysis of Wheat Tissue

The wheat leaves and roots were prepared for transmission electron microscopy (TEM) analysis immediately after harvest. The samples were prepared according to Lung et al. [27].

In addition, an elemental analysis was performed with a scanning electron microscope (SEM) HITACHI SU8230 cold field emission, operating at 30 kV and coupled with EDX (LIME-INCDTIM, Cluj-Napoca, Romania).

#### 2.4.5. Wheat Biomass and Soil Substrate Elemental Content Evaluation

Neutron activation analysis (NAA) at the pulsed fast reactor IBR-2 (FLNP JINR, Dubna, Russia) was used to determine the elemental content of the wheat biomass and soil substrate. The method of the elemental content determination was performed according to Lung et al. [27]. Certified standards were used for the analysis: trace elements in soil (2709), pine needles (1575a), calcareous soil (690CC), marine sediment (433), and trace elements in coal (1632c). The processing of the reference materials was performed in the same conditions as the samples, and the results are expressed on a dry weight basis.

### 2.5. Data Analysis

The values presented in this study represent the mean of three replicates ±SD (standard deviation). The data were analyzed using analysis of variance (ANOVA) to test for main effects and interactions, and terms were considered significant at *p* < 0.05.

In order to study and present the data, exploratory analysis methods were used within which two parameters were determined, namely mobility ratio of the elements from soil to plant [27] and mobility factor of the elements in soil substrate (*τ_i_*) [28].

In order to quantify the mass fraction of the element added or lost from the soil during the experiment relative to the mass of that originally present in the control material we used an open-chemical-system transport function defined by Chadwick et al. in 1990 [29]. Due to the fact that the experimental set-up was not as complicated as a geological one, the function was simplified in order to avoid such factors as physical collapse and dilation (strain). The equation was as follows:

$$\tau_{i}=\left({\left(C_{i}/C_{s}\right)}_{soil}/{\left(C_{i}/C_{s}\right)}_{soil\;control}\right)-1$$

where: *C* is the concentration of an element, the subscript *i* refers to the investigated supposed mobile element, and *s* to the reference element. A reference element is an element that is particularly stable in the soil, which is characterized by absence of vertical mobility and/or degradation phenomena and its concentration should not be anthropogenically altered. Typical reference elements used in many studies are Al, Sc, Fe, Mn, and Rb [28].

## 3. Results

### 3.1. NPs Characterization

#### 3.1.1. XRD Analysis

**Figure 1:**
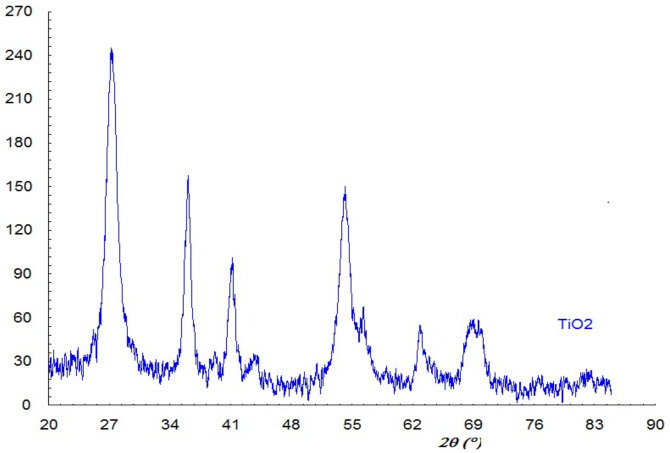
Diffractogram of TiO_2_ NPs.

The X-ray powder diffraction pattern for TiO_2_ NPs is shown in Figure 1. All diffraction peaks belong to pure Rutile TiO_2_ phase (PDF 89-8302), which is tetragonal, having the following unit cell parameter: a = 4.59 Å, b = 2.95 crystallite size was evaluated from an angle of 27.460 2θ using the Scherrer relationship, the obtained result being D = 57 Å.

#### 3.1.2. Morphological Analysis

**Figure 2:**
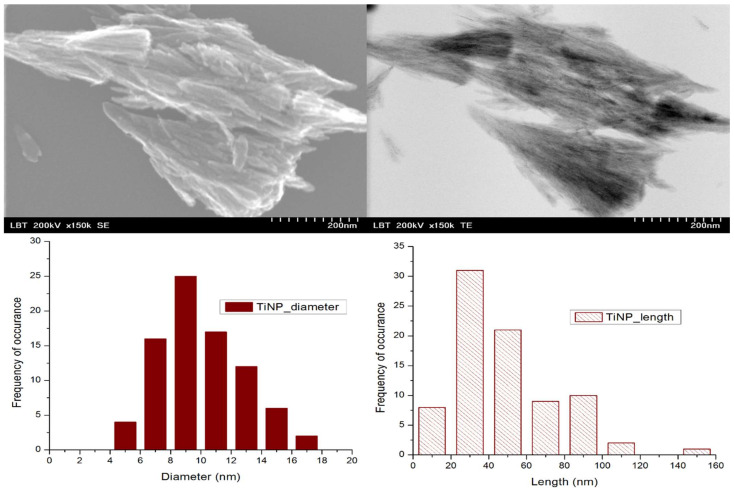
STEM micrographs of the TiO_2_ nanostructures and the diameter and length distributions.

The STEM analysis of the TiO_2_ nanostructures revealed a rod-like structure (Figure 2) with uniform distributions of the length and diameter, which might favor the uptake in the roots of *Triticum aestivum.*

#### 3.1.3. FT-IR Analysis

**Figure 3:**
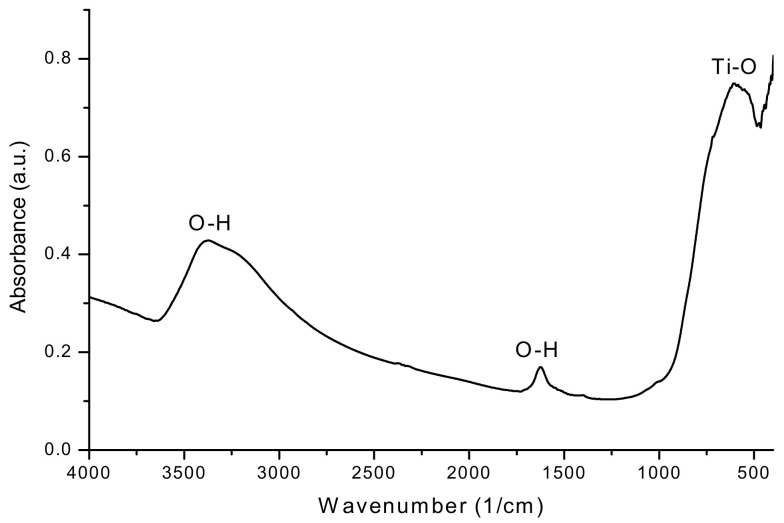
FT-IR spectrum of the analyzed nanoparticles.

The FTIR spectrum of TiO_2_ NPs is presented in Figure 3.

The following absorption bands are observed on the FTIR spectrum of TiO_2_ NPs: wide bands from 3374 cm^−1^, 1622 cm^−1^, and 1407 cm^−1^ correspond to stretching vibrations of hydroxyl groups and bending vibrations of the surface-adsorbed water molecules on the nanoparticles. The broad band between 750–480 cm^−1^, centered at 605 cm^−1^, can be attributed to the vibration of the Ti–O-Ti bonds in the TiO_2_ lattice [30,31].

### 3.2. Assessment of Plant Tissues

#### 3.2.1. Characterization of Assimilating Pigments

**Figure 4:**
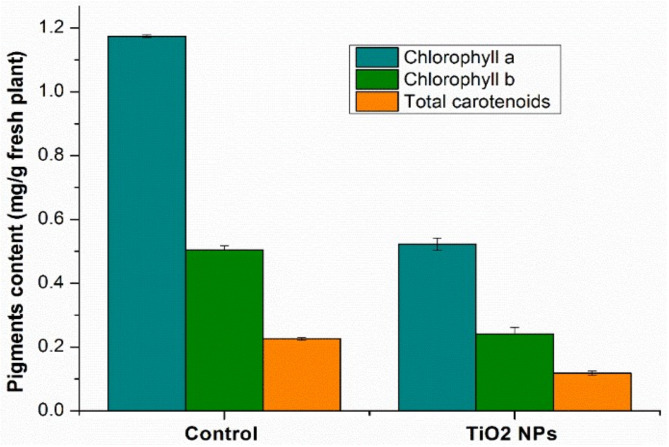
Comparative diagram of assimilating pigments. Each data point is the mean ± the standard error of the mean of three independent replicates experiments.

The chlorophyll plays an essential role in photosynthesis and any change in chlorophyll could affect plant growth. The carotenoids also play a vital role in defending plants against biotic and abiotic stress [32]. The amount of the assimilating pigments determined in the wheat samples analyzed is shown in Figure 4.

As can be seen in the wheat grown on amended soil with TiO_2_ NPs, the amount of pigments decreases compared to the control wheat. This decrease is 55% in the case of chlorophyll a, 52% in the case of chlorophyll b, and 48% in the case of total carotenoids. It has been observed that TiO_2_ NPs have positive effects on the photosynthetic apparatus in some plants, while in others they have negative effects, especially in algae [33]. Similar results were obtained by Dogaroglu and Koleli [34]. In another study developed by Dağhan et al. [35] on the impact of TiO_2_ NPs on the wheat in hydroponic medium, chlorophyll content decreased with increasing of TiO_2_ NPs concentration.

#### 3.2.2. Quantification of Total Polyphenolic Content

**Figure 5:**
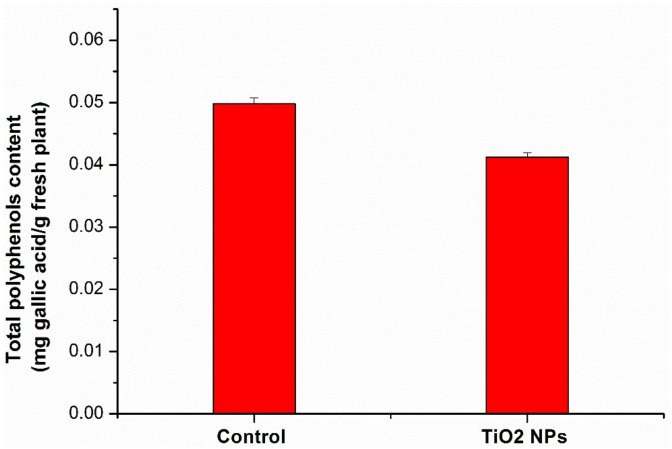
Total polyphenols content expressed as gallic acid equivalents in wheat samples. Each data point is the mean of the three independent replicates experiments ± the standard error.

The polyphenolic content from wheat samples is given in Figure 5.

Comparing the amount of polyphenols, it was found that in the case of wheat sample grown on amended soil with TiO_2_ NPs, this decreases (17%) compared to the control sample. Polyphenols are a large and diverse group of secondary metabolites involved in the defense mechanisms of plants against biotic or abiotic stress [36]. It is known that TiO_2_ NPs has a photocatalytic effect in phenolic components, such as lignin [37]. This could be the reason why the amount of polyphenols decreased. A decreased content of polyphenols was determined, also, in castor under the impact of Ag NPs [38], in *Azolla filiculoides* Lam in the presence of ZnO NPs [39].

#### 3.2.3. Determination of Total Antioxidant Capacity

**Figure 6:**
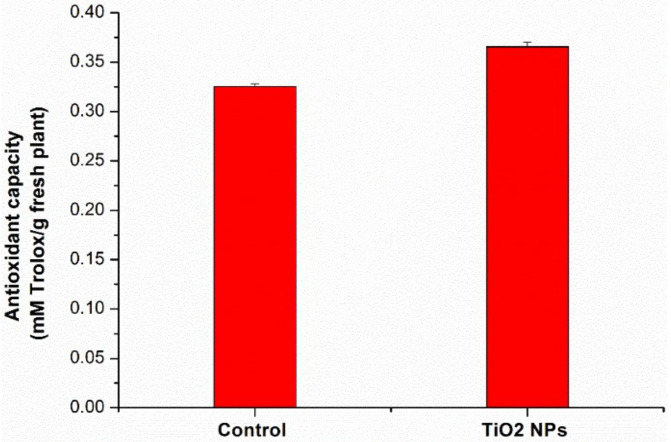
The antioxidant capacity of wheat extracts. Each data point is the mean ± the standard error of the mean of three independent replicates experiments.

The antioxidant capacity, expressed in mM Trolox equivalents, is presented in Figure 6.

The plants grown on amended soil with TiO_2_ NPs showed higher antioxidant capacity (12%) compared to control plants. To protect themselves from the effects of biotic or abiotic stress, plants maintain a balance by accumulating enzymatic and non-enzymatic components with a role in the defense system [40]. Studying the plants foliar treated with 0.75% suspension of TiO_2_ NPs, it was found that the antioxidant activity initially increased in the treated plants compared to the control and then decreased [41].

#### 3.2.4. TEM Analysis of Wheat Tissue

**Figure 7:**
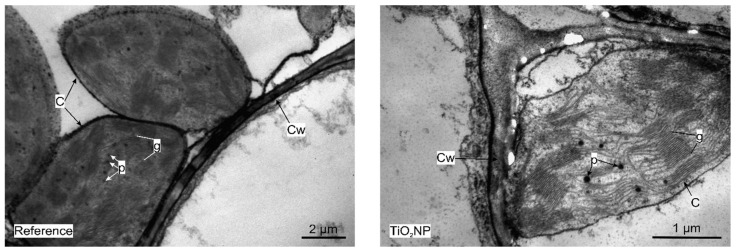
TEM micrographs of the untreated and TiO_2_ NP treated leaves. C = chloroplast, Cw = cell wall, g = grana, p = plastoglobuli.

**Figure 8:**
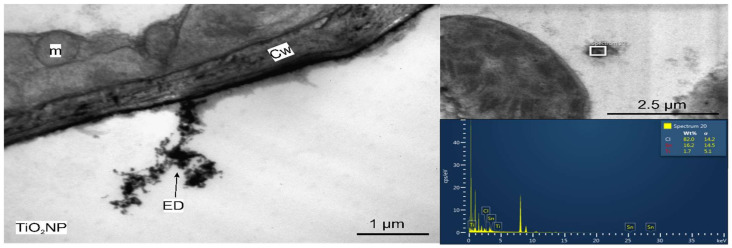
TEM micrograph and EDX analysis of the roots of TiO_2_ treated plants. Cw = cell wall ED = electron dense particles, m = mitochondria.

Leaf and roots of *Triticum aestivum* were analyzed through TEM to determine if there are any ultrastructural modifications. As compared to the untreated control, no significant alterations were observed on the leaves of TiO_2_ NPs treated plants (Figure 7). However, electron-dense nanostructures were detected in the cells of the roots, which were shown to have Ti in their composition, by the EDX analysis (Figure 8). These electron dense structures were absent in the leaves.

### 3.3. Soil and Plant Element Contents

#### 3.3.1. Soil Content and Mobility Factor in Soil

In both types of soil were determined 39 elements and their content is presented in Table 1.

Only two elements (Mg and Al) have significant decline of concentration level and ten elements (Cl, K, Fe, Co, Zn, As, Sr, Sn, Sb, and Au) have significant positive increase.

RD = 100 × (C_amanded_ − C_control_)/C_control_ Relative difference between concentration mean values. In our experiment, the open-chemical-system transport function *τ* defined above was used to follow the influence of amending soil with TiO_2_ NPs in comparison with the control soil. Typical reference elements used in many studies are Al, Sc, Fe, Mn, and Rb [28]. Among these elements, in our study, Sc had the smallest relative difference of the content in the experimental sample compared to the control: only 6.5% (Table 1). That is why we decided to use Sc as a reference lithogenic element.

If *τ_i_* < 0, reflecting the migration or net loss of elements relative to the control; if *τ_i_* > 0, the elements are relatively enriched or net obtained; and if *τ_i_* = 0, the behavior of elements in the profile is relatively stable [28].

For soil amended with TiO_2_ NPs, the majority of elements have *τ* values close to 0, between −0.2 and +0.2. Only Cl, Fe, Eu, Zr, and Sn have *τ* > 0.2. Mg, Al and Au present *τ* < −0.2. It might be concluded that addition of TiO_2_ NPs to the soil did not influence much the soil content.

#### 3.3.2. Plant Content and Interactions between Elements

From the analyzed elements, in wheat control were determined 32 elements (Na, Mg, Al, Cl, K, Ca, Sc, Cr, Mn, Fe, Co, Ni, Zn, As, Br, Rb, Sr, Mo, Sb, Cs, Ba, La, Ce, Nd, Sm, Eu, Tb, Tm, Yb, Ta, Th, and U), while in wheat grown in amended soil only 16 of them and Ti, V, Sn, Zr, Hf, W, and Au was not detected in any wheat samples. Ti was not detected in any of wheat samples, even in those grown on the soil amended with TiO_2_ NPs. This can be explained by the fact that in the total mass of applied NPs, only a small amount of NPs have both dimensions (diameter and length) less than 36 nm [42] thus being suitable for transfer from root to shoots (Figure 2) and the particles that have reached the root were practically mostly fixed in the deposits mentioned in Figure 8.

One of the reasons why only half of elements determined in control could be detected in plants grown with TiO_2_ NPs could be that exogenous application of NPs may change metal speciation in soil, which in turn could severely affect metal immobilization in the soil [43].

NPs phytotoxicity may affect the plant mineral nutrient uptake as a synergistic, antagonistic, or neutral. Mattiello and Marchiol [44] reported that for barley grown in soil treated with TiO_2_ NPs (0, 500, 1000, and 2000 mg kg^−1^), the mineral nutrition uptake was affected compared to the control treatment. Table 2 demonstrates the results of elemental analysis of plants grown in control and amended soil as well as literature data found for wheat leaves obtained from plants grown as control material in different studies. The Table focuses on those elements which could be detected in both types of wheat samples.

The content of some of elements (Al, Ca, Cl, Mg, and Rb) does not differ significantly in wheat grown with TiO_2_ NPs from control wheat.

The Sr concentration in plants is highly variable and is reported for different food and feed plants to range from about 10 to 1500 mg kg^−1^ DW [53,54]. Grodzinsky et al. [55] reported Sr content of 21 mg g^−1^ DW in control wheat leaves at early stages of plant development with a strong (three to four times) decrease with age. In our study, the Sr content in wheat grown with TiO_2_ NPs decreased about three times compared to control wheat, which is similar to data reported in literature [45,47,56]. Although Ba is not an essential component of plant tissues, it is often reported to be present in plants. Ba is regarded as a trace element ranging up to 100 mg kg^−1^ in leaves of cereal and legumes. In the case of wheat, some sources report that its content is higher in the leaf and stems, through the barium content of the wheat plant is small compared to other plants grown in the same soil [57]. Other researchers [56] report insignificant differences between Ba content in wheat roots and leaves. In our study, Ba content is similar to those reported in the literature [45,47] and decreased about five times in wheat grown with TiO_2_ NPs compared to control wheat.

Being at the same time an essential component of plants and potential contaminant, Zn absorption and transport, its biochemical functions, toxicity and tolerance in plants were widely studied. Zn content in plants varies considerably, reflecting different factors of various ecosystems and genotypes. The majority of available data refer to Zn content in wheat grains and shows a range from 22 to 33 mg kg^−1^ DW [54]. Zn content in grain ranges from 0 to 35%, up to 42% of the total Zn content during the maturation of the wheat plant [49]. Literature data reported for Zn content in wheat leaves vary from 50.5 to 120 mg kg^−1^ [47,51]. In our study, the Zn content is similar to the lower boundary of the range and increases about 1.4 times when wheat plants are grown in the presence of TiO_2_ NPs.

In spite of the fact that As is a constituent of most plants and its toxicity on wheat has been widely investigated during the last decades [58], no control values for wheat are scarce [47,52]. In our study, the As content decreased more than five times when TiO_2_ NPs was applied to soil.

Sb is considered a nonessential metal and is known to be easily uptaken by plants if present in soluble forms. The Sb content in wheat grown with TiO_2_ NPs decreased almost 18 times compared to the control. The control content in our study is similar to those reported in literature [45,52,59,60,61].

Mo is an essential micronutrient, but it is known that cereals require very little Mo and are rather insensitive to Mo deficiency [62]. Nevertheless, Mo deficiency causing the death of wheat seedlings during the winter season in southern China has become a problem limiting wheat yield [63]. In our study the use of TiO_2_ NPs leads to a significant (almost 19-fold) increase of Mo content in wheat. The Mo content in control wheat is similar to those reported elsewhere [47,64].

Although Br has long been known to be present in all plant tissues, it is still not known whether it is important for plant growth [65]. The Br concentration reported for leaves of wheat plants grown in control soils has large variation [60,62,65], and our results, both of the control and of the experimental samples, fall within this range [50]. Shtangeeva in 2017 [66] showed that Br concentration in young wheat seedlings grown in different liquid media (distilled water, spring water, nutrient solution of Hoagland) were similar. A 1.2–fold increase in Br was reported both in our experiment and by Shtangeeva when scandium bioaccumulation and its effect on uptake of macro- and trace-elements during initial phases of wheat growth was studied [67].

Numerous studies carried out on Mn uptake by plants and on Mn distribution among plant tissues have evidenced that Mn uptake is metabolically controlled, apparently in a way similar to that of other divalent cation species such as Mg^2+^ and Ca^2+^ [54], and its function is mainly related to the oxidation–reduction process. Several studies have been dedicated to wheat plants response to the application of Mn on wheat seeds and soil [68,69], while little information concerning Mn content in wheat is available [46,50]. Our data are close to those reported by Garnett and Graham [49] for steam of wheat grown in control soil within an experiment aimed to study the distribution and remobilization of iron and copper in wheat. The wheat gives the same response to the addition of TiO_2_ NPs as in Cu and Fe addition, i.e., a significant decrease of Mn content.

The mechanisms of Fe uptake and transport by plants and the essential role of Fe in plant biochemistry have received much attention. It has been shown that Fe content in wheat leaves grown in a Sc supplemented medium is lower than in control samples while response to the addition of Eu, Ca, and a Ca + Eu mixture is insignificant [56,61]. In our study, amendment with TiO_2_ NPs led to a significant (almost 13-fold) decrease of Fe in wheat plants from 1350 to 105 mg kg^−1^. Lyu et al. reported that Ti may act antagonistically with Fe resulting in Ti toxicity in plants [70].

The nutrient ratios may provide valuable information about the influence of external factors on a plant. In particular, K/Na ratios are important characteristics of the plant physiological state [71]. As reported [56], K/Na ratio in leaves of control wheat seedlings before and after transfer to soil was 58 and 177, respectively. In seedlings grown with Ca, Eu, and a mixture of Ca and Eu, the K/Na ratios were 66%, 77%, and 89% of the control value, respectively. In our study, the ratio increased up to 594 dues to a significant uptake of K and decrease of Na. This may indicate a serious imbalance induced by amendment with TiO_2_ NPs.

All living organisms tend to a chemical balance that is a basic condition for their proper growth and development. Interactions of chemical elements also have similar importance to deficiency and toxicity in the physiology of plants. Interactions between chemical elements may be both antagonistic and synergistic, and their imbalanced reactions may cause a real chemical stress in plants. Antagonism occurs when the combined physiological effect of two or more elements is less than the sum of their independent effects, and synergism occurs when the combined effects of these elements is greater. These interactions may also refer to the ability of one element to inhibit or stimulate the absorption of other elements in plants [54].

It seems that the addition of TiO_2_ NPs to soil may intensify the antagonisms between some elements, as was already mentioned [54], or it may itself act as a releaser.

It was found that only As, Ba, Br, Fe, K, Mn, Mo, Na, Sb, Sr, and Zn differed significantly in wheat samples grown on modified soils, compared to control wheat. The content of some elements varied relatively slightly; the content of Br and Mn increased and decreased by 1.22 times, respectively, while other elements (K, Na, Fe, and Sb) varied by more than 10 times. We observed the greatest difference between experimental samples and control for Mo—an almost 19-fold increase in the content.

**Figure 9:**
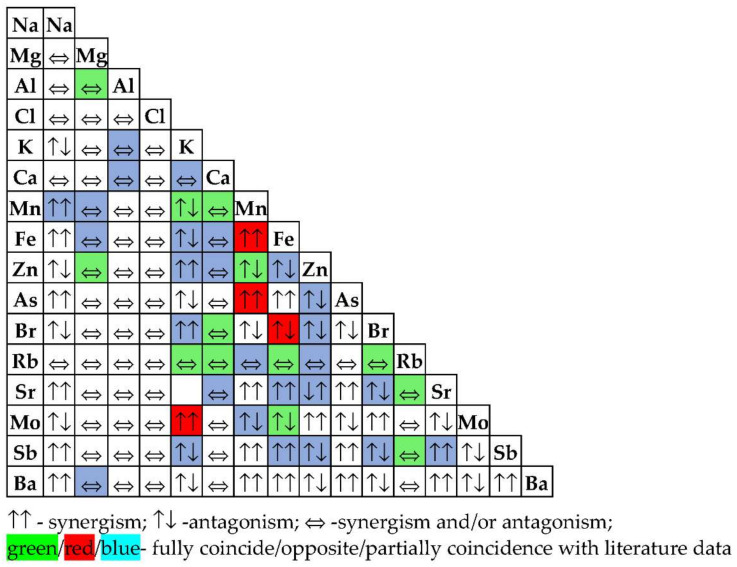
The comparison of our results with the data in the literature.

Kabata-Pendias and Pendias, in 2001 [54] gave a well-structured summary interaction between major and trace elements in plants. From data provided by Shtangeeva et al. [62] and Shtangeeva and Ayrault [56] concerning the elemental response of wheat seedlings to addition of Sc, Ca, Eu, and Ca + Eu in substrate we also made some indirect conclusions about the interaction of elements. Figure 9 shows the comparison of our results with the data in the literature. The results that found similar statements in the literature are presented in green, while those that are contrary to the literature are marked in red. Those results that partially coincide with literature are colored in blue.

The content of Mg, Al, Cl, Ca, and Rb did not vary significantly when TiO_2_ NPs was added to the substrate; therefore, it could be considered that their interaction with the rest of the elements cannot be clearly defined. It might be both synergistic and/or antagonistic.

Nevertheless, in literature it is also mentioned that Al and Zn may be both antagonist and synergic to Mg as well as Mn, Br, and Rb to Ca. Most of the conclusions about elemental interactions in plants are contradictory in different sources, suggesting that the interaction is a process highly dependent on external factors, such as the addition of different chemical elements to the soils.

#### 3.3.3. Soil to Plant Transfer

The mobility ratio was calculated in order to establish the transfer of each determined element from soil to plant [72]. Plants can act like accumulators (MR > 1), like excluders (MR < 1), or they can have an indifferent behavior to some elements (MR ~1) for a 20% variation (from 0.8 to 1.2) [73].

Wheat plants (control and those grown on amended soil) are excluders of Al, As, Ba, Ca, Fe, Mg, Mn, Na, Sb, Sr, and Zn

Both plant samples have a very high accumulator behavior for Cl, the highest value belonging to the control wheat. In case of K, the wheat sample grown on amended soils with TiO_2_ NPs has very significant accumulator behavior, while the control wheat sample is indifferent to K.

The control wheat accumulates Rb, but the wheat samples grown on amended soil with TiO_2_ NPs is indifferent to Rb.

### 3.4. Correlation between the Element Content and Phenolic Compound Concentration, Assimilating Pigments Concentration, Antioxidant Capacity in Plants

**Figure 10:**
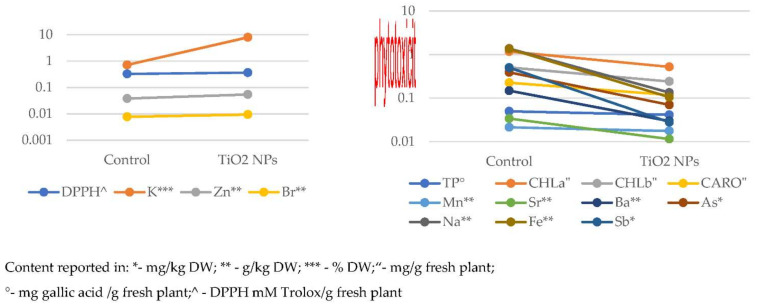
Graphical representation of the variation of the content of bioactive compounds and chemical elements.

As can be observed in Figure 10, the significant decrease of TP, CHL a, CHL b, and CARO is accompanied by the decline of Na, Mn, Fe, As, Sr, Sb, and Ba content, while an increase of DPPH correlates with enrichment in K, Zn, Br, and Mo.

A similar behavior for K and Zn in relation to all biologically active parameters was previously reported when wheat plants were grown in soil amended with different types of CuO NPs [27]. The addition of CuO NPs and TiO_2_ NPs results in the same correlation between most of the elements common to the two experiments (Na, Fe, As, Sr, Sb, Ba, and Mo) and CHL a, CHL b, and DPPH.

The addition of CuO NPs and TiO_2_ NPs results in the same correlation between most of the elements common to the two experiments (Na, Fe, As, Sr, Sb, Ba, and Mo) and CHL a, CHL b, and DPPH.

Dağhan et al. have been shown a significant increase of Zn, decrease of chlorophyll, Fe, and K content in wheat with the increase of TiO_2_ NPs supply in hydroponic medium, while variation of Mn content was not statistically relevant [35]. Dogaroglu and Koleli also reported that TiO_2_ NPs supply led to a significant decrease in chlorophyll content of wheat shoots [34].

Available studies on the impact of TiO_2_ NPs on plants argue for the beneficial effects that NPs’ application may have on plant growth and productivity. Feizi et al. [74] reported that nanosized TiO_2_ treatments in an appropriate concentration (about 10 ppm) could accelerate the seed germination and seedling growth of wheat in comparison to bulk TiO_2_. Ullah et al. [75] revealed that application of 50 mg kg^−1^ of TiO_2_ NPs significantly enhanced the root and shoot length of wheat. The increase of nutrients content in the shoots, for Ca, Cu, Al, and Mg with 50 mg kg^−1^ TiO_2_ NPs treatment reflected improvement in plants’ growth and grain’s quality. The activities of antioxidant enzymes (SOD and POD) were enhanced in shoot with increasing concentration of TiO_2_ NPs up to 50 mg kg^−1^. Similar to these results, TiO_2_ NPs increased the content of Cu, Fe, and Mn in basil plants [76]. Other researchers [77] showed that 30 mg L^−1^ of TiO_2_ NPs is the optimum concentration for significant increase in germination, root and shoot length, fresh biomass, and vigor index of parsley (*Petroselium crispum*).

These studies note that the optimal concentration of NPs is relatively low and at higher concentrations negative effects begin to be noticed. The increase of nanosized TiO_2_ concentrations after 10 ppm decreased shoot and seedling lengths. The lowest shoot length was achieved at 100 ppm nanosized TiO_2_ [74]. The activities of antioxidant enzymes were decreased if TiO_2_ NPs concentration increased from 50 to 100 mg kg^−1^ [75]. Above 30 mg mL^−1^, Dehkourdi and Mosavi [77] reported that the chlorophyll contents of parsley decreased significantly. In basil leaves, nano-TiO_2_ significantly reduced CAT activity at 250 mg kg^−1^, APOX activity and Fe content at all concentrations, compared with controls [76].

Recently, studies have begun to appear in the literature on the effect of applying NPs on plants grown in soils contaminated with various elements, but the impact of potential NPs contamination has not been studied either. Our study attempted to cover some knowledge gaps by applying to plants a high amount of NPs in order to track the effects of accidental soil contamination with TiO_2_ NPs.

It is possible that a decrease of chlorophyll can be connected to the capacity of nanoparticles to penetrate through seeds. Low content of NPs can activate the embryo, increasing the germination rate [78], while a high NPs content could enhance the activity of chlorophyllase which has an important role in chlorophyll breakdown [77].

We suppose that in our study, the increase in DPPH has the same origin as that supposed by Tan et al. [76], in their study, i.e., the blockade of absorption channels by nanoparticles, causing stress responses due to limited absorption of essential nutrients. This is partly confirmed by the decrease in Na, Mn, and Fe, while the increase in Zn and K content calls it into question. Servin et al. [79] also found that TiO_2_ NPs (500 mg kg^−1^) present in soil promoted K accumulation up to 35% more in treated cucumber than in the control plant.

Clearly, there is a relationship between TiO_2_ NPs and plant nutrient uptake, but plant species have a specific mechanism for nutrient uptake. Unfortunately, there is not enough data available to clearly define it.

Practically all available literature sources refer to studies focused on the impact of deficiency or surplus of single or paired elements to certain plants without a multi-element approach. The conclusions concerning the correlation between increase or decline of the elemental content and bioactive compounds are quite contradictory. More multielemental studies are required to explore the robust mechanisms underlying NPs-mediated trace elements immobilization in soil and uptake by plants.

## 4. Conclusions

This article aimed to determine the effect of TiO_2_ NPs on bioactive compounds, elemental content, and ultrastructure of wheat leaves. It was found that the amount of assimilating pigments and polyphenols decreased compared to the control wheat, while the antioxidant capacity was increased in the treated plants compared to control ones.

The addition of TiO_2_ NPs to soil is followed by a significant response of the plant to the stress factors, revealed by an increase in antioxidant capacity, which goes along with decrease in chlorophyll, total polyphenols, and carotenoids, and follows the significant positive (K, Zn, Br, and Mo) and negative (Na, Mn, Fe, As, Sr, Sb, and Ba) variation of content, respectively. This study provides some information about the impact of TiO_2_ NPs on the content of certain chemical elements in wheat, which have received less attention so far, but which, at first glance, show a fairly high variability (Mo, Br, As, Ba, Sb, and Sr). The elemental interactions in plants and soil are highly dependent on the external factors, such as amending with NPs, and only a multi-element approach can elucidate the nature of some of them.

## Figures and Tables

**Table 1:** Experimental and statistical results on soil (mg/kg ± SD).

Element	C_control soil_	C_amanded soil_	RD (%)	*τ_i_*	Element	C_control soil_	C_amanded soil_	RD (%)	*τ_i_*
Al *	19,800 ± 990	16,400 ± 820	−17.2	−0.22	Nd	7.2 ± 1.5	7.6 ± 1.2	5.6	−0.01
As *	5.4 ± 0.38	6.6 ± 0.46	22.2	0.15	Ni	9.2 ± 0.92	10.7 ± 0.96	16.3	0.09
Au *	0.22 ± 0.068	0.014 ± 0.004	−93.6	−0.94	Rb	26.6 ± 4.8	29.0 ± 5.2	9.0	0.02
Ba	317 ± 22	372 ± 19	17.4	0.10	Sb *	2.46 ± 0.180	2.70 ± 0.098	9.8	0.03
Br	11.6 ± 0.35	10.8 ± 0.32	−6.9	−0.13	Sc	3.1 ± 0.09	3.3± 0.10	6.5	
Ca	71,100 ± 9240	67,000 ± 8710	−5.8	−0.11	Sm	1.60 ± 0.31	1.64 ± 0.15	2.5	−0.04
Ce	17.3 ± 1.5	17.8 ± 1.2	2.9	−0.03	Sn *	1.25 ± 0.075	1.72 ± 0.103	37.6	0.29
Cl *	455 ± 50	1170 ± 105	157.1	1.42	Sr *	110 ± 9.7	139 ± 7.7	26.4	0.19
Co *	4.0 ± 1.16	4.8 ± 1.14	20.0	0.13	Ta *	0.306 ± 0.095	0.340 ± 0.105	11.1	0.04
Cr	21.4 ± 1.70	25.0 ± 2.00	16.8	0.10	Tb	0.203 ± 0.033	0.220 ± 0.025	8.4	0.02
Cs	1.47 ± 0.062	1.56 ± 0.047	6.1	0.00	Th	2.57 ± 0.14	2.60 ± 0.12	1.2	−0.05
Eu	0.087 ± 0.003	0.18 ± 0.06	106.9	0.94	Ti	1660 ± 150	1810 ± 145	9.0	0.02
Fe *	7440 ± 1340	10,700 ± 535	43.8	0.35	Tm	0.123 ± 0.036	0.133 ± 0.025	8.1	0.02
Hf	1.77 ± 0.088	1.93 ± 0.096	9.0	0.02	U	1.17 ± 0.045	1.12 ± 0.047	−4.3	−0.10
K *	6010 ± 361	7240 ± 450	20.5	0.13	V	28 ± 1.40	26 ± 1.80	−5.7	−0.11
La	9.5 ± 1.0	9.4 ± 0.75	−1.1	−0.07	W	2.35 ± 0.753	2.43 ± 0.728	3.4	−0.03
Mg *	21,000 ± 840	16,400 ± 656	−21.9	−0.27	Yb	0.610 ± 0.097	0.680 ± 0.102	11.5	0.05
Mn	650 ± 39	604 ± 36	−7.1	−0.13	Zn *	83 ± 3.32	102 ± 4.50	22.9	0.15
Mo	0.800 ± 0.245	0.790 ± 0.250	−1.3	−0.07	Zr	57 ± 7.7	78 ± 14.2	36.8	0.29
Na	2610 ± 183	2380 ± 190	−8.8	−0.14					

* Differences between element concentrations in the control and amended soils were significant *(p* < 0.05).

**Table 2:** Experimental and literature data for wheat content (mg/kg ± SD) and mobility ratio.

Element	C_control wheat_	C_TiO_2_ NPs_	MR_c_	MR_TiO_2_ NPs_	Literature Data on Wheat Leaves		
					Min	Max	References
Al	63 ± 3.8	73 ± 4.4	0.0032	0.0045	5.9 ± 5.5	29.2 ± 3.9	[45,46]
As *	0.39 ± 0.035	0.07 ± 0.006	0.072	0.011	0.01 ± 0.004	<0.09	[47,48]
Ba *	148 ± 10.4	29.7 ± 2.67	0.47	0.08	9.8 ± 11.5	29 ± 18	[45,47]
Br *	7.7 ± 0.23	9.4 ± 0.28	0.66	0.87	8.4 ± 4	111 ± 10	[49,50]
Ca	3330 ± 800	2720 ± 650	0.047	0.041	50 ± 8	5600 ± 1500	[48,51]
Cl	15,800 ± 1264	14,500 ± 1160	34.7	12.4	5520 ± 4110	27,000 ± 3000	[47,51]
Fe *	1350 ± 67.5	105 ± 12.6	0.18	0.01	160 ± 20	225 ± 28	[45,49]
K *	7140 ± 641	79,600 ± 720	1.19	10.99	19,000 ± 5460	65,700 ± 52,500	[47,52]
Mg	1810 ± 72	1700 ± 68	0.09	0.10	79 ± 13	2200 ± 500	[45,51]
Mn *	21.4 ± 1.3	17.6 ± 1.01	0.033	0.029	2 ± 0.4	44.1 ± 7.8	[46,50]
Mo *	0.107 ± 0.036	2.02 ± 0.627	0.13	2.56	0.47 ± 0.11	-	[47]
Na *	1370 ± 55	134 ± 5.4	0.52	0.06	300 ± 100	5100 ± 330	[49,50]
Rb	41 ± 7.4	29 ± 5.2	1.54	1.00	10 ± 5.8	88 ± 22	[47,48]
Sb *	0.5 ± 0.04	0.028 ± 0.004	0.20	0.010	0.02 ± 0.01	0.98 ± 0.14	[45,52]
Sr *	34 ± 3.4	11.5 ± 1.38	0.31	0.08	5.17 ± 4.79	15 ± 7	[45,47]
Zn *	38 ± 2.28	54 ± 3.24	0.46	0.53	50.5 ± 8.3	120 ± 6	[47,50]

* Difference between element concentrations in the control wheat and wheat grown with TiO_2_ NPs were significant (*p* < 0.05). MR = C_wheat_/C_soil_ Mobility Ratio.

## Data Availability

Not applicable.
